# Extra and intracranial metastasis as an initial presentation of testicular cancer

**DOI:** 10.11604/pamj.2018.31.141.16434

**Published:** 2018-10-25

**Authors:** Carlos Eduardo Salazar-Mejía, Marusia González-Villarreal

**Affiliations:** 1 Centro Universitario Contra el Cáncer, University Hospital “Dr. José Eleuterio González” and Faculty of Medicine, Universidad Autónoma de Nuevo León, Monterrey, Nuevo León, México; 2 Internal Medicine Department, University Hospital “Dr. José Eleuterio González” and Faculty of Medicine, Universidad Autónoma de Nuevo León, Monterrey, Nuevo León, México

**Keywords:** Testicular cancer, young men, brain metastasis

## Image in medicine

A 24-year-old man presented with a 9-month history of a progressively increased painless left testicle. One month before admission, a round tumor appeared on his right forehead; this was associated with a persistent frontal headache of moderate intensity. The tumor progressively increased and was accompanied by altered consciousness characterized by stupor and confusion, which motivated his being taken to the emergency room. On initial evaluation, he had a Glasgow Coma Score of 13 with a proportionate left hemiparesis and a left pyramidal syndrome. The left testicle was increased in size, indurated, painless to palpation, and associated with a large left hydrocele. A brain MRI was performed that showed a heterogeneous lobulated intra and extracranial right frontal lesion. The intracranial component was vascularized and hemorrhagic, 8.2 x 7.1 x 6.8 cm with partially defined borders and vasogenic edema which shifted the midline 30 mm to the left and infiltrated the superior sagittal sinus. This predisposed right subfalcine and uncal herniation as well as left hydrocephalus. Extension studies showed metastatic lung and liver lesions. Laboratory studies revealed an alpha-fetoprotein level of 1210 ng/mL with a normal β-hCG level (1.11 mIU/mL). A diagnosis of poor-risk germ-cell tumor was made and a chemotherapy scheme was planned; however, the patient died due to acute neurological deterioration 48 hours after admission.

**Figure 1:**
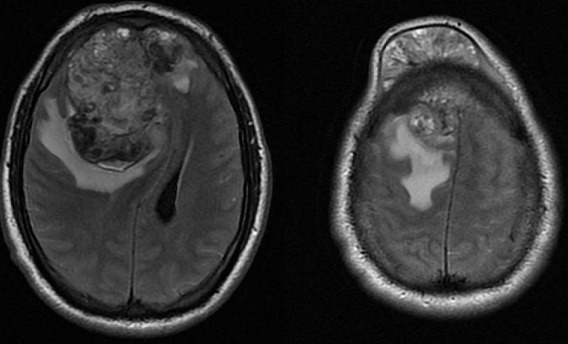
T2 MRI sequence that shows a large metastatic intra and extracranial right frontal lesion

